# Effect of a single-dose denosumab on semen quality in infertile men (the FITMI study): study protocol for a randomized controlled trial

**DOI:** 10.1186/s13063-022-06478-4

**Published:** 2022-06-22

**Authors:** Sam Kafai Yahyavi, Rune Holt, Li Juel Mortensen, Jørgen Holm Petersen, Niels Jørgensen, Anders Juul, Martin Blomberg Jensen

**Affiliations:** 1 Group of Skeletal, Mineral, and Gonadal Endocrinology, Department of Growth and Reproduction, Copenhagen University Hospital - Rigshospitalet, Copenhagen, Denmark; 2 International Center for Research and Research Training in Endocrine Disruption of Male Reproduction and Child Health (EDMaRC), Copenhagen University Hospital - Rigshospitalet, Copenhagen, Denmark; 3 Section of Biostatistics, Faculty of Health Sciences, University of Copenhagen, Copenhagen, Denmark; 4 Department of Growth and Reproduction, Copenhagen University Hospital - Rigshospitalet, Copenhagen, Denmark; 5 Department of Clinical Medicine, University of Copenhagen, Copenhagen, Denmark; 6 Division of Bone and Mineral Research, HSDM/HMS, Harvard University, Boston, USA

**Keywords:** Denosumab, Male infertility, Impaired semen quality, Randomized controlled study

## Abstract

**Background:**

Infertility is a common problem globally and impaired semen quality is responsible for up to 40% of all cases. Almost all infertile couples are treated with either insemination or assisted reproductive techniques (ARTs) independent of the etiology of infertility because no medical treatment exists. Denosumab is an antibody that blocks RANKL signaling and inhibition of testicular RANKL signaling has been suggested to improve semen quality in a pilot study. This RCT aims to assess whether treatment with denosumab can improve spermatogenesis in infertile men selected by serum AMH as a positive predictive biomarker. This paper describes the design of the study.

**Methods/design:**

FITMI is a sponsor-investigator-initiated, double-blinded, placebo-controlled 1:1, single-center, randomized clinical trial. Subjects will be randomized to receive either a single-dose denosumab 60 mg subcutaneous injection or placebo. The study will be carried out at the Department of Growth and Reproduction, Copenhagen University Hospital, Rigshospitalet, Copenhagen. The primary outcome of the study is defined as the difference in sperm concentration (millions pr. mL) one spermatogenesis (80 days) after inclusion.

**Discussion:**

We describe a protocol for a planned RCT aimed at evaluating whether treatment with denosumab can improve the semen quality in infertile men selected by using serum AMH as a positive predictive biomarker. The results will provide evidence crucial for future treatment in a patient group where there is a huge unmet need.

**Trial registration:**

Clinical Trials.gov NCT05212337. Registered on 14 January 2022.

EudraCT 2021–003,451-42. Registered on 23 June 2021.

Ethical committee H-21040145. Registered on 23 December 2021.

**Supplementary Information:**

The online version contains supplementary material available at 10.1186/s13063-022-06478-4.

## Administrative information

The order of the items has been modified to group similar items (see http://www.equator-network.org/reporting-guidelines/spirit-2013-statement-defining-standard-protocol-items-for-clinical-trials/).

**Table:** 

Title	Effect of a single-dose denosumab on semen quality in infertile men (the FITMI study): study protocol for a randomized controlled trial
Trial registration	Clinical Trials, NCT05212337. Registered on 14 January 2022 EudraCT: 2021–003,451-42. Registered on 23 June 2021 Ethical committee: H-21040145. Registered on 23 December 2021
Protocol version	Version 3.2 updated May 11 2022
Funding	XY Therapeutics
Author details	All authors except the statistician are primarily associated with the Department of Growth and Reproduction, Copenhagen University Hospital—Rigshospitalet, Copenhagen, Denmark. See the title page for further details
Name and contact information for the trial sponsor	Martin Blomberg Jensen, MD, DMSc Group of Skeletal, Mineral, and Gonadal Endocrinology, Department of Growth and Reproduction, Rigshospitalet, University of Copenhagen, Denmark E-mail: blombergjensen@gmail.com
Role of sponsor	This is a sponsor-investigator-initiated study supported financially by the company ‘XY Therapeutics’ that obtain full access to the anonymous data. The design of the study has been performed by investigators and ‘XY Therapeutics’ but the sponsor-investigator maintains authority over all aspects of the trial, including, design, management, interpretation of results, and publication

## Background

### Introduction and rationale

Infertility is a common problem globally and impaired semen quality is responsible for up to 40% of all cases. Despite the high prevalence there are currently only very limited treatment options to improve semen quality for infertile men [1, 2]. Instead, almost all infertile couples are treated with inseminations or assisted reproductive techniques (ARTs) independently of the etiology of infertility [3–7]. ARTs are very successful but expensive and associated with a significant treatment burden of the female partner due to the invasive methodology and the need for hormonal treatment often for several months.

RANKL is a ligand for the receptor activator of nuclear factor κB (RANK), and their pathway plays a prominent role in the regulation of bone metabolism. The binding of RANKL to RANK on osteoclast precursors induces osteoclast maturation and activation, thereby stimulating bone resorption [8], and regulates cell cycle i.e., proliferation, differentiation, and apoptosis [9, 10]. Osteoprotegerin (OPG) is a secreted decoy receptor that controls RANKL-RANK interaction by binding RANKL and thereby inhibits activation of RANK [11] and preventing osteoclast differentiation and activation [12].

Denosumab, a drug used in millions of patients worldwide under the trade names Prolia® and Xgeva®, inhibits the RANKL pathway and is used to treat osteoporosis and bone metastases [10]. The drug’s mechanism of action inhibits RANKL and thus inhibits bone resorption through reduced osteoclast activation. This reduces the loss of bone mineral density (BMD), which reduces the risk of bone loss and thereby the risk of fracture and osteoporosis [13, 14]. Denosumab has been shown in several clinical studies to be a safe and effective drug in both women and men and has been in clinical use in both sexes for many years [15–18]. As denosumab has a teratogenic effect, pharmacokinetic studies in both monkeys and healthy men were performed before approval of the drug as a treatment for osteoporosis in men. These studies showed that denosumab concentration in semen does not pose a risk to the fetus during sexual intercourse with the pregnant woman and therefore is safe to use for the suggested infertility indication as there is no risk of fetal transmission [19, 20].

### Our research

Among others, our research group has demonstrated the role of vitamin D in male reproduction by using functional animal studies supported by a randomized clinical trial [7, 21, 22]. Further investigations revealed that several bone factors such as Runx2, Osterix, FGF23, and in particular RANKL are expressed in the testis [5, 6, 23–26]. We found expression of RANKL in Sertoli cells, the receptor RANK in the testicular germ cells, and OPG in the peritubular cells in mice and human tissue [26]. The presence of the RANKL system in these distinct testicular cell types indicates a possible direct effect on spermatogenesis.

We therefore investigated the effect of denosumab in human testicular germ cell lines as well as in human testicular tissue ex vivo “hanging drop” cultures [26]. Denosumab treatment in both cases increased the proliferation of the germinal cells. These studies confirmed that denosumab treatment in vitro has a possibly beneficial effect on sperm production by reducing apoptosis in the germ cells. To further investigate this in vivo, we injected the natural RANKL inhibitor OPG into mice daily for 14 days and compared them with their controls. Here, we found a significantly increased testicular weight, increased thickness of germinal cell epithelium, and markedly higher sperm production [26]. This prompted a human pilot study of 12 infertile men who besides infertility were healthy young men under 40 years of age. The men were treated with one 60 mg dosage of denosumab subcutaneous (s.c.). The pilot study showed that as a group, the men’s sperm production had increased 80 days after treatment. However, there was a large variation and 60% of men experienced an increase between 100–600% in sperm counts. The rest of the participants did not appear to benefit from the treatment [26]. To validate putative biomarkers, a placebo = controlled RCT was conducted in 100 infertile men with severe male infertility (Denosumab and male infertility: a RCT. ClinicalTrials.gov Identifier: NCT03030196). In this study, active treatment with 60 mg denosumab s.c. was compared to placebo treatment. Here we identified serum AMH as a predictive biomarker. Importantly, we did not experience any serious adverse reactions nor severe hypocalcemia, or increased abortion rate in the female partners of the patients. Data from this study has not been published yet.

In general, infertility in men is a heterogeneous disease but by identifying serum levels of AMH as a positive predictive biomarker, it seems we can select the group of infertile men where treatment with denosumab most likely will increase their semen quality.

### Objective

This RCT aims to assess whether treatment with denosumab can improve semen quality in infertile men selected by serum AMH as a positive predictive biomarker. This paper describes the design of the study.

## Methods/design

### Trial design and setting

**Fig. 1:**
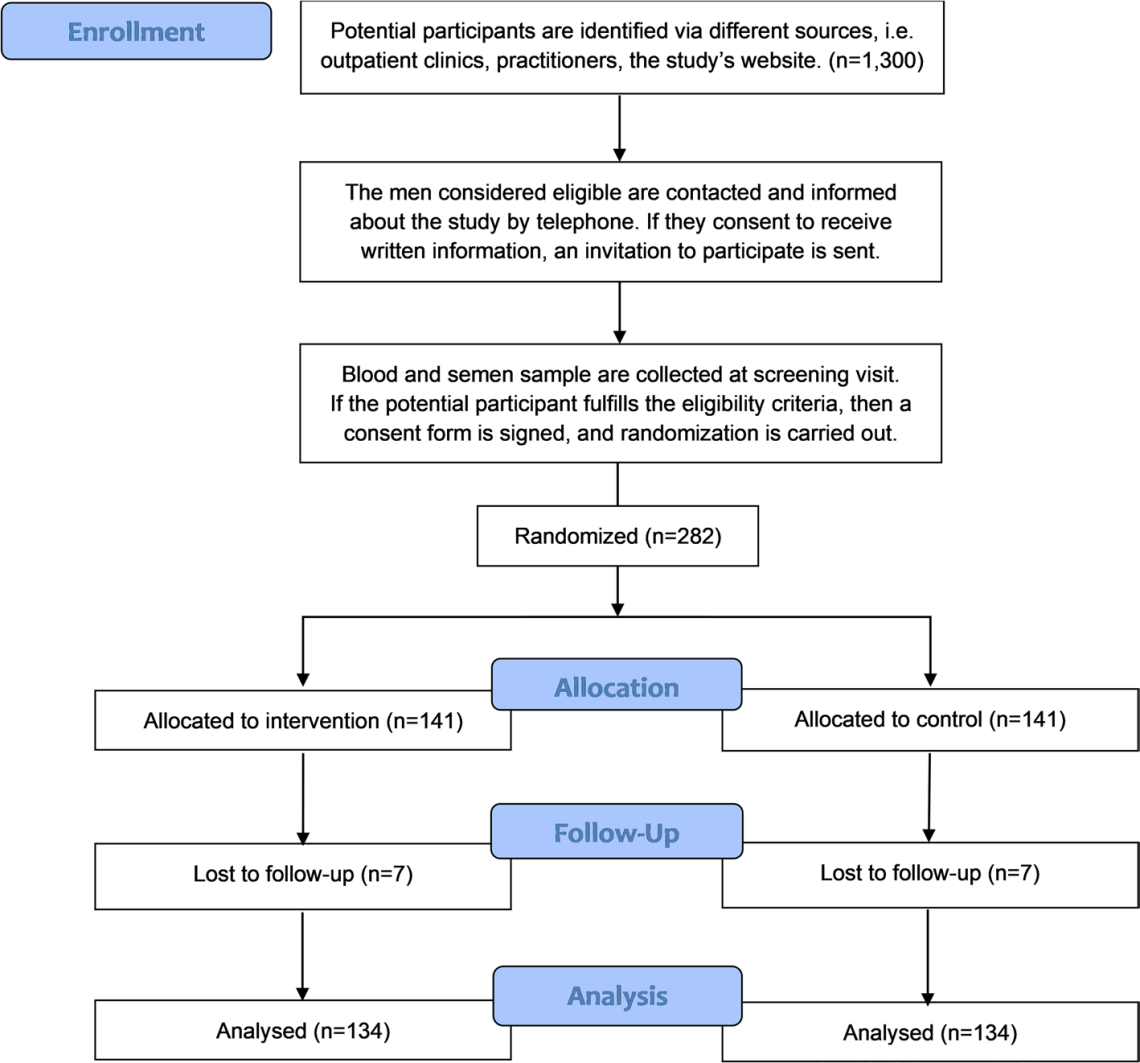
The expected flow diagram of the progress through the study (CONSORT). Figure legend: An expected CONSORT diagram showing the expected flow of FITMI

FITMI is a single-center, sponsor-investigator-initiated, placebo-controlled, double-blinded randomized clinical phase 2 trial, designed as a superiority trial with two parallel groups and a primary endpoint defined as the difference in sperm concentration (million pr. mL) between the denosumab and placebo arm. Following successful completion of screening procedures, subjects will be randomized in a 1:1 fashion to receive either denosumab 60 mg s.c. or a placebo. The study will be carried out at the Department of Growth and Reproduction, Copenhagen University Hospital—Rigshospitalet, Copenhagen. The SPIRIT reporting guidelines have been used for reporting [27] and a CONSORT diagram has been shown in Fig. 1.

### Inclusion criteria

Eligible participants will be infertile men ≥ 18 years and < 60 years of age with a sperm concentration ≤ 20 million pr. mL and serum AMH levels ≥ 38 pmol/L. These inclusion criteria were selected based on the two previous intervention studies in infertile men. To be included, the participants must have appropriate Danish or English language skills and give written informed consent.

### Exclusion criteria

Potential participants will be excluded from participation for the following reasons: Chronic diseases, defined as diagnosis where signs, symptoms, and treatment imply an expected long duration and lack of a cure, such as diabetes mellitus, thyroid disorders, autoimmune diseases such as arthritis, vasculitis or inflammatory bowel disease and osteoporosis. A sperm concentration < 2 million pr. mL. Also, men with current or previous malignancies, or at potential risk of testicular cancer after baseline examination and ultrasound will be excluded. Furthermore, men with hypocalcemia, vitamin D deficiency or impaired kidney function at baseline, defined as ionized calcium of < 1.18 mmol/L or albumin corrected calcium < 2.17 mmol/L or total calcium < 2.14 mmol/L, serum vitamin D (25OHD) levels < 25 nmol/L or eGFR < 60 mL/min/1,73 m^2^ will also be excluded. Finally, insufficient dental status, vasectomy, semen volume < 0.9 mL or hypersensitivity to latex, denosumab, or to any of the excipients (acetic acid, sodium hydroxide, Sorbitol (E420), Polysorbate 20) will be excluded.

### Withdrawal

Participants will be withdrawn from the study after randomization if they meet one or more of the following criteria: occurrence of malignancies, calcium disorders, parathyroid disorders, thyroid disorders, diabetes or other endocrine disorders that require hormone treatment, fever defined as a temperature above 38.5 °C for more than 24 h. Furthermore, treatment with drugs such as chemotherapies, diuretics, calcium channel blockers, Salazopyrines, corticosteroids, TNF-alpha blockers, Cellcept, Imurel, Protopic, or methotrexate. Also, if a testicular biopsy or other genital surgery is performed during the trial period or if the patient is admitted with temporary or permanent organ failure of either liver, heart, kidney, lung, gastrointestinal system, or urogenital system. Lastly, if participants withdraw consent or there is a breach of blinding on the subject.

### Recruitment, screening, and enrollment

The participants will be recruited via different channels, but mainly from the outpatient clinic at our department. In addition, we will contact and visit public and private fertility clinics, endocrinology departments, and private practices in Copenhagen and encourage sites to refer patients directly to our study. We will also draw attention to the study via media. A website where potential participants can read about the study will also be created. When we are contacted by a potential participant, he will be briefly informed about the study and offered to receive detailed material. As a starting point, we will only invite infertile men with a sperm concentration that is expected to meet the eligibility criteria. In a screening visit, we will explain the trial in detail and if he is still interested and agrees to participate, informed consent will be signed. Also, at the screening visit, the participant must produce a semen sample and have a blood sample taken where we measure serum AMH, vitamin D, calcium, and creatinine. In the event where a participant has had ejaculation (including intercourse and masturbation) less than 2 days before the visit, the semen sample will not be included in the final analysis. Participants will be included in the trial regardless of ethnicity and social status, as long as they fulfill the eligibility criteria. Informed consent is always obtained by an investigator or under the supervision of an investigator. Patients will be reminded of their appointments by text message to promote participant retention and a better follow-up. Potential drop-outs will be contacted by one of the investigators to evaluate the reason which will be noted.

### Randomization

To ensure a completely randomized distribution according to the eligibility criteria, participants will be divided into four groups based on their sperm concentration and serum AMH. Separation thresholds will be sperm concentration at 9 million pr. mL and serum AMH at 50 pmol/L. In this way, the four groups will consist of participants with sperm concentration 2.0–9.0 million pr. mL and serum AMH 38–50 pmol/L (Group A), sperm concentration 2.0–9.0 million pr. mL and serum AMH > 50 pmol/L (Group B), Sperm concentration 9.1–20 million pr. mL and serum AMH 38–50 pmol/L (Group C) and sperm concentration 9.1–20 million pr. mL and serum AMH > 50 pmol/L (Group D). The sperm concentration used, and serum AMH thresholds are estimated based on previously conducted experiments with infertile men, whereby we get 4 groups with the same number of expected participants in each group.

### Blinding process

The blinding itself will be prepared centrally by a local pharmacy (Glostrup Apotek). Treatment drug will be allocated as block randomization in the four arms described above, in blocks of eight unknown to investigators. The pharmacy will be responsible for labeling both placebo and intervention treatment. The medicine will arrive in closed boxes with labels on the outside, indicating each unique random number. A nurse who has not had contact with the participant will handle the box and transfer the contents, either a prefabricated syringe containing 1 mL denosumab 60 mg or a prefabricated ampoule containing isotonic saline, to a new syringe that thereby is blinded for everyone else. Unfortunately, this step is necessary as matching placebo only can be obtained through the pharmaceutical company Amgen, who declined to provide this. To ensure that the trial will be double-blind only the nurse who prepares the injection will be aware of whether participants have received denosumab or saline but will not have any contact with the participant. Another nurse will pick up the prepared syringe and thus be blinded and inject the participant with the medicine (denosumab or placebo). This means the allocated trial medication will be blinded to the participant, the clinical staff caring for the participant, the investigators, the outcome assessors, the data manager, the statistician conducting the analyses, and the writing committee when drafting the abstract for the primary publication. The empty packaging will be put back in the box, which is sealed with tape. All used boxes will be stored in a locked room. If an individual breach of code is required, the box with the associated unique random number can be found and opened.

### Outcome measures

The primary outcome of the study is defined as the difference in sperm concentration (million pr. mL) between the denosumab and placebo arm. For this purpose, the average sperm concentration of two semen samples delivered on day 80 and day 83 after inclusion is used.

Secondary endpoints include the difference in semen quality (total sperm count, total number of motile sperm, percentage of motile sperm, total number of progressive motile sperm and percentage of progressive motile sperm, total number of morphologically normal sperm, and percentage of morphologically normal sperm) between the denosumab and placebo arm. Also, differences in pregnancies achieved spontaneously or by IUI before day 180, live births where pregnancy is achieved spontaneously or at IUI before day 180, and the number of live births where pregnancy is achieved by artificial insemination (IVF and ICSI) before day 180. Furthermore, the difference in the number of miscarriages throughout the trial period and difference in serum levels of reproductive hormones (testosterone, estradiol, FSH, LH, AMH, Inhibin B, SHBG, and INSL3) on day 80. All the mentioned secondary endpoints will be quantified as the difference between groups.

As exploratory endpoints, we will look at the change in testosterone/estradiol ratio, changes in serum levels of RANKL and OPG, changes in RANKL, OPG, AMH, and Inhibin B in seminal fluid, and modifications of mineral homeostasis in serum and seminal fluid (measurements of calcium, albumin, phosphate, magnesium, zinc, bicarbonate, PTH, 25OHD, and creatinine/eGFR).

### Timeline

Table 1 shows the timeline for the participants of the trial. If the participant fulfills the eligibility criteria, he is invited to the day 1 visit. Here they will provide another semen sample, have a blood sample drawn, and finally receive a subcutaneous injection of either denosumab 60 mg or placebo. They will also be handed out supplementation containing 180 tablets of 10 μg vitamin D and 400 mg calcium. On day 80, another semen sample is provided, another blood sample is drawn, and a clinical follow-up visit is scheduled with a nurse. Three days later on day 83, the participant delivers the final semen sample. This is the last physical visit for the participant. On day 180, the participants will receive an electronic questionnaire focusing on pregnancies and adverse events. If they do not respond to this form, they will be contacted by telephone. If they have achieved pregnancy before day 180, there will be sent an additional electronic questionnaire on day 450 regarding pregnancy complications, birth complications as well as data on the child (gender, birth weight, birth length, and whether the child is healthy). Finally, 2 years after *last participant last visit*, we will extract data from The Danish National Patient Register, to follow up on unexpected long-term side effects.

**Table 1:**
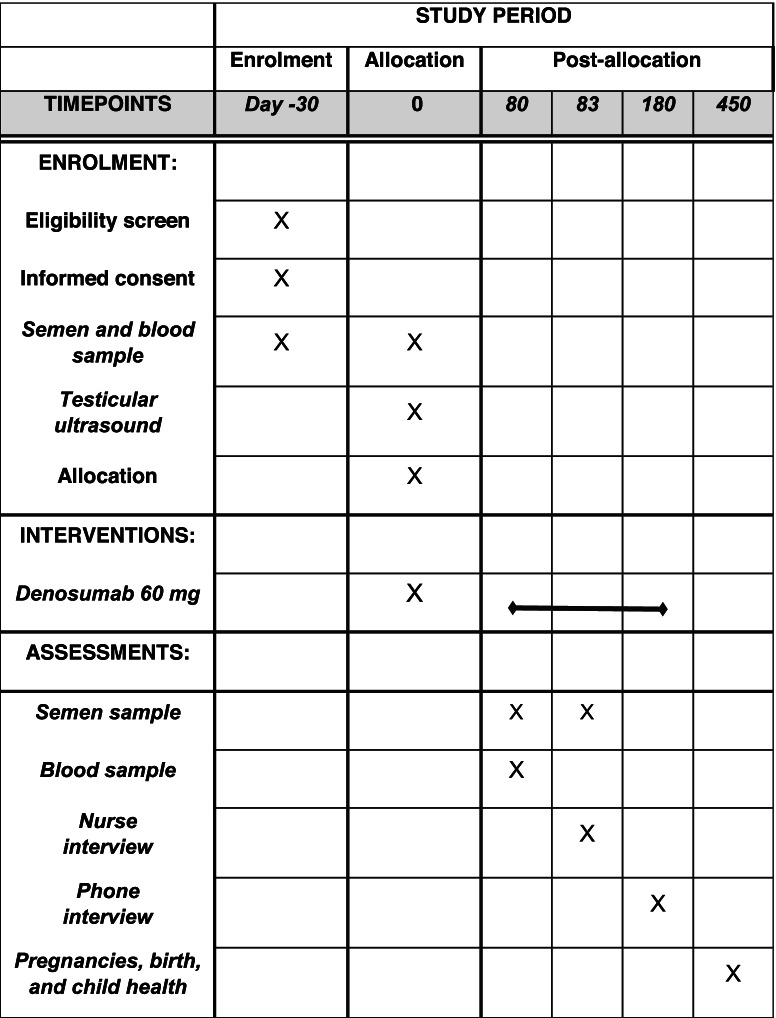
The expected timeline flow for patients participating in the trial

A SPIRIT figure showing the expected timeline for the participants. If the participant fulfills the eligibility criteria, he is invited to the day 1 visit. Here they will provide another semen sample, have a blood sample drawn, and finally receive a subcutaneous injection of either denosumab 60 mg or placebo. On day 80 and day 83, the participants deliver the final semen samples. This is the last physical visit, while they will receive an electronic questionnaire asking about pregnancies and adverse events 6 months later. If they have achieved pregnancy before day 180, there will be sent an additional electronic questionnaire on day 450 regarding pregnancy complications, birth complications as well as data on the child

### Ethical considerations

The trial will be conducted in accordance with the principles of the Declaration of Helsinki and compliance with the protocol approved by the competent authority and Ethics Committee, and according to GCP standards [28]. No deviation from the protocol will be implemented without the prior review and approval of the regulatory authorities except where it may be necessary to eliminate an immediate hazard to the trial participants. All participants will be informed of potential adverse effects, and that they can leave the trial at any point without any consequences. Denosumab is proven to be safe in several randomized studies and is already approved by both FDA and EMA. There was no effect on sperm DNA fragmentation index (DFI) or abortions in the first two clinical trials performed by our research group so there are no known safety concerns. All side effects will be closely monitored and reported. Furthermore, in general, there is currently no medical treatment to improve male fecundity which makes ARTs the primary option for infertile couples. This is a treatment where women must undergo fertility treatment and often receive several hormonal drugs associated with side effects. This is a significant ethical issue which in our view fully justifies the mild potential side effects caused by denosumab.

## Data analysis

### Analysis population

Data will be analyzed using intention-to-treat (ITT) principles. When applying the ITT principle, all randomized participants will be analyzed in the groups to which they were originally allocated, regardless of whether they received the intended treatment or whether a protocol violation or protocol deviation occurred. Participants who withdraw consent for the use of their data will not be included in any analysis and withdrawal of consent will be reported. A CONSORT flow diagram of participants will be presented in the study.

### Sample size

With the power to avoid a type II error set to 80% (1-β) at a two-sided 5% significance level 141 men in each of the investigation arms are needed to detect a difference in sperm concentration of 45% between the intervention and placebo group in the primary outcome. A group-sequential design allows one interim analysis at half target recruitment. We estimate to screen 1,300 infertile men as around 30% will meet the eligibility criteria and 70–75% will agree to participate in the trial. The calculations are based on the intra-individual variation in sperm concentration when including infertile men with sperm concentrations between 2 and 20 million pr. mL. We expect that the placebo group will have a post-trial sperm concentration of 11 million pr. mL while the denosumab group will have 16 million pr. mL with a maximum SD of 15. At an SD of 10, the same effect can be demonstrated by the inclusion of 170 men which is the basis for the interim analysis.

### Statistics and underlying assumptions

A detailed statistical analysis plan is attached as an additional file (see Additional file 1). In brief, descriptive statistics, including unadjusted baseline and follow-up averages for the entire study population, as well as baseline and follow-up averages for the four subgroups will be presented. The primary analysis will be a covariance analysis in which day 80 measurements are analyzed, initially as crude values but also regressed on baseline (including treatment assignment and subsequently AMH assignment). This will correctly take into account the grouped randomization scheme as well as the correlation between the day 80 and baseline measurements. Baseline is defined as the average of day − 30 and day 1 and day 80 is defined as the average of day 80 and day 83, unless abstinence time is < 2 days or high fever which will result in exclusion of data. In both cases, data will be transformed as necessary to meet model assumptions. Subgroup analyses, e.g. for the group > 9 mill/mL semen concentration will be performed. These analyses will not have the nominal type I error and will be interpreted as “hypothesis generating” results. Subsequently, subgroup analyses, e.g. for the group > 9 mill/mL sperm concentration, low versus high baseline, FSH, Inhibin B and testis size, and men with and without cryptorchidism and varicocele will be performed. Also, all regression analyses will be tested for major interactions between each covariate and the intervention variable. For each combination, we will test whether the interaction term is significant and assess the effect size. When reporting a potentially relevant clinically significant effect, due diligence will be exercised because of the risk of type I errors when performing multiple tests.

### Interim analysis

One interim analysis of safety and efficacy data will be evaluated when 170 patients have delivered their final semen sample at day 83. These assessments will be made by an independent statistician with focus on primary outcomes and serious adverse events. The interim analysis will be carried out using a two-sided significance test with the O’Brien–Fleming alpha spending function [29] and a type I error rate of 5 percent. To adjust the type 1 error rate for multiplicity, we will use the O’Brien-Fleming method in a group-sequential design, resulting in an alpha level for the interim analysis at 0.01 and the final analysis at 0.046. The statistician will review the protocol and monitoring guideline, evaluate the attempts to recruit participants and participants’ risk, and, based on interim analyses, make recommendations to sponsor and investigators as to whether to continue the study or end it.

## Data management

### Software

All data will be entered as an electronic database in REDcap. REDcap is a worldwide online system developed specifically for non-commercial clinical research to significantly reduce data entry and study management errors to improve data fidelity. The system is offered to researchers in the capital region of Denmark. Our data is thus protected by a security code and only accessible for registered users. Besides informed consent, there will be no data in paper form. The study will be notified to the Research and Innovation Unit, The Capital Region of Denmark (Videnscenter for Dataanmeldelser). The law on the processing of personal data will be complied with. After completion of the trial, data will be anonymized and shared with “XY Therapeutics” who can use the data for possible approval by the EMA and FDA authorities. The sharing of data with “XY Therapeutics” will be reflected in participant information and informed consent so that it is clear for the participants.

### Monitoring

The trial will be externally monitored by the national Good Clinical Practice (GCP) unit at Bispebjerg hospital. A monitoring plan will be conducted before trial initiation. The frequency of onsite monitoring will depend on compliance with the protocol, the number of enrolled participants, and the quality of data handling. There will be mandatory monitoring before the trial and at least three times during the trial. The GCP will monitor inclusion and exclusion criteria, consent obtained in all subjects according to legislation, and data included in the eCRF. The investigators will be responsible for all data in the eCRFs. All personal information about potential and enrolled participants are collected in the eCRF and only accessible by relevant users which secures confidentiality before, during, and after the trial.

### Follow-up data and handling of missing data

Missing data will be minimized by performing repeated monitoring of data entry into electronic case report forms (our eCRFs). In this way, we will be able to monitor the extent of missing data and intervene if necessary. Hence, we do not anticipate that there will be any significant number of missing values.

### Safety

Denosumab is a drug already used in millions of patients worldwide. Common adverse effects associated with denosumab are abdominal pain, constipation, musculoskeletal pain, skin rash, and infection. Rare adverse effects are symptomatic hypocalcemia, osteonecrosis of the jaw, atypical femoral fracture, and anaphylaxis [30]. The side effect profile is better than most competing drug treatments used to treat osteoporosis, and denosumab is well tolerated by patients with competing diseases such as kidney disease, in contrast to, i.e., zoledronic acid. In FITMI, Adverse events (AE), serious adverse events (SAEs), serious adverse reactions (SARs), and suspected unexpected serious adverse reactions (SUSARs) will be recorded in the intervention period and will be reported to the relevant authorities according to guidelines from GCP and the Danish Medicines Agency. To avoid symptomatic hypocalcemia participants with vitamin D deficiency and/or hypocalcemia are excluded and concomitant treatment with 10 μg vitamin D and 400 mg calcium is given to all participants for 180 days. Furthermore, to ensure that the handling of safety is independent of sponsor and investigators, a safety committee will be set up. The members of the safety committee will be four senior medical doctors who are leading experts in bone diseases and reproductive diseases in men. All serious incidents or side effects will be referred to the safety committee within 72 h and they will decide on the need for code breaches.

### Biological specimens

All samples are stored in a research biobank so all analyzes can be run simultaneously to avoid excessive inter-assay variation. The biobank consists of − 20° freezers located at Rigshospitalet in a locked room. A maximum of 5 years after the start of the experiment (01.01.2027), the samples will be transferred to a biobank at Hvidovre hospital and stored at − 80° for future research. The samples are stored here for another 25 years, i.e., until 01.01.2047. If a participant withdraws his or her consent to participate, all collected biological material will be destroyed.

### Dissemination plans

The results of the trial will be submitted for publication in international peer-reviewed journals and submitted for presentation at international conferences. Authors SKY and RH will be shared as first authors, MBJ will be the last author, and other researchers contributing will be listed as co-authors. There are no plans to grant public access to participant-level datasets or any other individual patient data after the trial is completed, but requests can be submitted directly to the corresponding author.

## Discussion

Infertility is a common problem globally and male factor with impaired semen quality is responsible for up to half of all cases [31, 32]. Despite the high prevalence there exist no medical treatment options to improve semen quality for most men [1, 2]. Contrary, most treatments are targeted at the woman, as she must undergo hormonal treatment to optimize conditions for ART. These treatments are often associated with many side effects including serious side effects and a high economic cost for the couple and/or the health care system. Today, we are treating females for a male disease.

The FITMI study is therefore highly relevant and is the first RCT designed to evaluate the effectiveness of denosumab as a first-in-class treatment for male infertility in men with impaired semen quality and high serum AMH. In addition to the relatively large sample size, follow-up data will be provided, an intention-to-treat analysis will be carried out, and the statistician performing the statistical analyses will be blinded to the allocation. Furthermore, baseline data will be collected to allow a comparison of the two groups, data on future pregnancies will be documented, and data on safety and serious adverse events will be reported. The results will provide evidence crucial for future treatment in a patient group where treatment options are sparse at best.

### Trial status

Participants are currently being recruited. The recruitment period is estimated to run from January 2022 to December 2023, with follow-up until September 2025. Further information will be available at http://www.fitmi.dk and clinicaltrial.gov (NCT05212337).

## Supplementary Information

**Additional file 1: Appendix.** A statistical analysis plan (SAP) for the FITMI study.

## Data Availability

Data sharing does not apply to this article, because no datasets were generated or analyzed during the present study.
