# Supplementary material for: Effect of a single-dose denosumab on semen quality in infertile men (the FITMI study): study protocol for a randomized controlled trial
Source: Trials. 2022 Jun 22;23:525. doi: 10.1186/s13063-022-06478-4 (PMC9214471; doi:10.1186/s13063-022-06478-4)
Supplement: Supplementary file 1 — Additional file 1: Appendix. A statistical analysis plan (SAP) for the FITMI study. [file 13063_2022_6478_MOESM1_ESM.docx]

**Appendix:
A statistical analysis plan (SAP) for the FITMI study**

**Section 1: Administrative information**

| Title {1} | Effect of a single-dose Denosumab on semen quality in infertile men (the FITMI Study): A detailed statistical analysis plan |
| --- | --- |
| Trial registration {1b} | ClinicalTrials.gov, NCT*****. Registered on 21 January 2022. EudraCT: 2021-003451-42. Registered on 23 June 2021. Ethical committee: H-21040145. Registered on 23 December 2021. |
| Protocol version {2-4} | SAP Version 1 updated January 20 2022 - No revision Main Protocol Version 1.3 updated January 21 2022 |
| Author details {5} | All authors except the statistician are primarily associated with the Department of Growth and Reproduction, Copenhagen University Hospital - Rigshospitalet, Copenhagen, Denmark. See the title page for further details. |
| Signatures {6} | Sam Kafai Yahyavi Rune Holt  Martin Blomberg Jensen Jørgen Holm Petersen |

**Section 2: Introduction
Background and rationale**
The background and rationale for our study has been described in the study protocol. In brief, infertility is a common problem in the world and the male factor in form of impaired semen quality is responsible for up to 40% of all cases (1,2). Despite this, there exist no medical treatment options to improve semen quality for most of these men (3,4). Contrary, most treatments are targeted on the partner, as the female has to undergo several hormonal cycles to optimize conditions for ART. These treatments are often associated with high economical cost, and many side effects for the women. The FITMI Study is therefore a highly relevant study and is the first RCT designed to evaluate the effectiveness of Denosumab as a first-in-class medical treatment for male infertility.

In accordance with good clinical practice as defined by the Helsinki Declaration (5) and the International Conference on Harmonization of Good Clinical Practice (ICH-GCP) (6), this appendix describes the statistical analysis plan (SAP) for FITMI. Guidelines recommend that clinical trials should be analyzed according to a prespecified plan to prevent selective outcome reporting bias (7) and data-driven analysis results (8,9) and ultimately, minimize the risks of bias.

Therefore, in the following we present a SAP for the FITMI trial that is predefined and developed before the database is locked in and data analysis has commenced. Participant recruitment started in January 2022 and is still on-going at this point in time.

**Study objective**FITMI is a randomized controlled trial that aims to assess whether treatment with Denosumab can improve semen quality in infertile men selected by serum AMH as a positive predictive biomarker.
**Section 3: Study Methods**
**Trial design**FITMI is a single-center, sponsor-investigator-initiated, placebo-controlled, double-blinded randomized clinical trial. Patients who are eligible according to the inclusion and exclusion criteria and who consent to participate after having received written and verbal information about the trial, are randomized in a 1:1 fashion to receive either Denosumab 60 mg s.c. injection or placebo. A total of 282 participants, 141 men in each of the investigation arms, is needed to detect a difference in sperm concentration of 45% between intervention and placebo group. The study will be carried out at the department of Growth and Reproduction, Copenhagen University Hospital, Rigshospitalet, Copenhagen. The Statistical Analysis Plan (SAP) Checklist reporting guidelines have been used for reporting (10).
 **Randomization**To ensure a completely randomized distribution according to the eligibility criteria, participants will be divided into four groups based on their sperm concentration and serum AMH. Separation thresholds will be sperm concentration at 9 million pr. mL and serum AMH at 50 pmol/L. In this way, the four groups will consist of participants with sperm concentration <9 million pr. mL and serum AMH <50 pmol / L (Group A), sperm concentration <9 million pr. mL and serum AMH ≥ 50 pmol/L (Group B), Sperm concentration ≥ 9 million pr. mL and serum AMH <50 pmol/L (Group C) and sperm concentration ≥ 9 million pr. mL and serum AMH ≥ 50 pmol/L (Group D). The sperm concentration used and serum AMH thresholds are estimated on the basis of previously conducted experiments with infertile men, whereby we get 4 groups with same number of expected participants in each group.

**Sample size**With the power to avoid a type II error set to 80% (1-β) at a two-sided 5% significance level for the primary outcome, 141 men in each of the investigation arms is needed to detect a difference in sperm concentration of 45% between intervention and placebo group. A group-sequential design allows one interim analysis at half target recruitment. We estimate to screen 1,300 infertile men as around 30% will meet the eligibility criteria and 70-75% will agree to participate in the trial.

**Interim analysis**One interim analysis of safety and efficacy data will be evaluated when 170 patients have completed the study. These assessments will be made by an independent statistician and focus on reporting baseline data and primary outcomes. The interim analysis will be carried out using a two-sided significance test with the O’Brien–Fleming alpha spending function (11) and a type I error rate of 5 percent. To adjust the type 1 error rate for multiplicity, we will use the O’Brien-Fleming method in a group-sequential design, resulting in an alpha level for the interim analysis at 0.01 and for the final analysis at 0.046. The statistician will review the protocol and monitoring guideline, evaluate the attempts to recruit participants and participants’ risk, and, on the basis of interim analyses, make recommendations to investigators as to whether to continue the study.

**Timeline**A structured timeline has been described in the main article. All outcomes will be analyzed collectively after unblinding of data.

**Section 4: Statistical principals**

**Analysis population**Data will be analyzed according to Good Clinical Practice guidelines and using intention-to-treat (ITT) principles. In this way, we include all randomized patients in the groups to which they were originally allocated, regardless of whether a protocol violation or protocol deviation occurs. Patients who withdraw consent for the use of their data will not be included in any analysis and withdrawal of consent will be reported.

**Statistics and underlying assumptions**Descriptive statistics, including unadjusted baseline and follow-up averages for the entire study population, as well as baseline and follow-up averages for the four subgroups will be presented. The primary analysis will be a covariance analysis in which day 80 measurements are analyzed, initially as crude values but also regressed on baseline (including treatment assignment and subsequently AMH assignment). This will correctly take into account the grouped randomization scheme as well as the correlation between the day 80 and baseline measurements. Baseline is defined as the average of day -30 and 0 and Day 80 is defined as the average of day 80 and 83, unless abstinence time is <2 days or high fever which will result in exclusion of data. In both cases, data will be transformed as necessary to meet model assumptions. Subgroup analyses, e.g. for the group >9 mill/mL semen concentration will be performed. These analyses will not have the nominal type I error and will be interpreted as "hypothesis generating" results. Subsequently, subgroup analyses, e.g. for the group >9 mill/mL sperm concentration, low versus high baseline, FSH, Inhibin B and testis size and men with and without cryptorchidism and varicocele will be performed. Also, all regression analyses will be tested for major interactions between each covariate and the intervention variable. For each combination, we will test whether the interaction term is significant and assess the effect size. When reporting a potentially relevant clinically significant effect, due diligence will be exercised because of the risk of type I errors when performing multiple tests.

**Blinding of the statistician**The detailed analysis plan was written in strict concordance with the trial protocol approved by the regulatory authorities (ethics committee and Danish medical agency) prior to recruitment initiation. The entire statistical analysis plan will be uploaded to clinicaltrials.gov before the trial is finalized (before the database will close). In general, all data managers, statistician, and those drawing conclusions will be blinded to treatment allocation. Analyses will be done prior to breaking of the randomization code (i.e. analysis will be performed between “A” and “B”). The principal investigators (SKY and RH) and the study sponsor (MBJ) will conjointly perform all the data analyses according to this plan, except the interim analyses which will be performed by an independent statistician, who is not an investigator of this trial. After the conclusions have been drawn, blinding will be broken, and the final manuscript will be based on the correct pre-written protocol.

**Section 5: Trial population**

**Eligibility criteria**Eligible participants will be infertile men ≥ 18 years and < 60 years of age with a sperm concentration between 2 and 20 million pr. mL and serum AMH levels > 38 pmol/L. The participants must have appropriate Danish or English language skills and give written informed consent.

Potential participants will be excluded from participation for the following reasons: Chronic diseases, defined as diagnosis where signs, symptoms, and treatment imply an expected long duration and lack of a cure, such as diabetes mellitus, metabolism disorders, osteoporosis, colitis, etc. Also, men with current or previous malignancies, or at potential risk of testicular cancer after baseline examination and ultrasound will be excluded. Furthermore, men with hypocalcemia at baseline, defined as ionized calcium of < 1,18 mmol/L or albumin corrected calcium < 2,17 mmol/L or total calcium < 2.14 mmol/L, serum vitamin D (25OHD) levels < 25 nmol/L or eGFR < 60 mL/min/1,73 m^2^ will be excluded. Finally, insufficient dental status, vasectomy, semen volume < 0.9 mL or hypersensitivity to latex, Denosumab, or to any of the excipients (acetic acid, sodium hydroxide, Sorbitol (E420), Polysorbate 20) will be excluded.

**Recruitment, screening, and enrollment**The participants will be recruited via different channels, but mainly from the outpatient clinic at our department. In addition, we will contact and visit public and private fertility clinics, endocrinology departments, and private practices in Copenhagen and encourage sites to refer patients directly to our study. We will also draw attention to the study via social and print media. A website where you can read about the study and sign up for screening will also be created. When we are contacted by a potential participant, he is briefly informed about the study and offered to receive detailed material. As a starting point, we will only invite infertile men with a sperm concentration that is expected to meet the eligibility criteria. In a screening visit we will explain the trial in detail and if he is still interested and agrees to participate, informed consent will be signed. Also, at the screening visit, the participant must make a semen sample and have a blood sample taken where we measure serum AMH, vitamin D, calcium, and creatinine. Trial participants will be included in the trial regardless of ethnicity and social status, as long as they fulfill the eligibility criteria. Informed consent is always obtained by an investigator or under the supervision of an investigator.
 **Baseline patient characteristics**The following baseline characteristics of the study population will be summarized within each randomized group:

- Included men, n (%)
- Age, years
- Ethnicity, Caucasian or other, n (%)
- Body mass index, kg/m^2^
- Children, n (%)
- ARTs, n (%)
- Duration of infertility, months
- Testicular size, mL
- Semen at baseline
  - Abstinence, days
  - Semen volume, mL
  - Sperm concentration, 10^6^/mL
  - Sperm motility, %
  - Progressive sperm motility, %
  - Sperm morphology, %
  - RANKL, pmol/L
  - OPG, pmol/L
- Blood sample at baseline
  - FSH, U/L
  - LH , U/L
  - AMH, pmol/L
  - Inhibin B, pg/mL
  - Testosterone, nmol/L
  - Estradiol, pmol/L
  - RANKL, pmol/L
  - OPG, pmol/L

Baseline characteristics in the table will be presented as means with standard deviation (95% confidence intervals) or medians with interquartile range (IQR), depending on distribution of data.

**Section 6: Analysis**

**Study outcomes**

The primary endpoint of the study is defined as:

- Difference in sperm concentration (million pr. mL) between the intervention and placebo arm. For this purpose, the average sperm concentration of two semen samples delivered on day 80 and day 83 after inclusion is used.

Secondary endpoints include:

- Difference in sperm quality (total sperm count, total number of motile sperm, percentage of motile sperm, total number of progressive motile sperm and percentage of progressive motile sperm, total number of morphologically normal sperm and percentage of morphologically normal sperm), between the intervention and placebo arm.
- Differences in pregnancies achieved spontaneously or at IUI before day 180
- Live births where pregnancy is achieved spontaneously or at IUI before day 180
- Number of live births where pregnancy is achieved by artificial insemination (IVF and ICSI) before day 180.
- Difference in the number of miscarriages throughout the trial period.
- Difference in serum levels of reproductive hormones (FSH, LH, AMH, Inhibin B and INSL3) and sex hormones (Testosterone, estradiol and SHBG) on day 80.

As exploratory endpoints, we will look at the following endpoints, quantified as the between-group difference of the given endpoint on day 80.

- Change in testosterone/estradiol ratio
- Change in serum levels of RANKL and OPG
- Changes in the semen levels of RANKL, OPG, AMH and Inhibin B
- Modifications of mineral homeostasis in serum and semen (measurements of calcium, phosphate, magnesium, zinc, bicarbonate and citrate).

**Follow-up data and handling of missing data**Missing data will be minimized by performing repeated monitoring of data entry into electronic case report forms (our eCRFs). In this way, we will be able to monitor the extent of missing data and intervene if necessary. Hence, we do not anticipate that there will be any significant number of missing values. As recommended, we will describe reasons why outcome data are missing in the main study manuscript (12). Furthermore, we will compare explanatory variables between all participants randomized to intervention groups (including those with missing outcomes), and also between participants in the intervention groups, where outcomes are reported. This is done to identify imbalances between groups due to missing outcome data (12). **Sub-group analysis**

The sub-groups of the study are defined as:

- Baseline sperm production: a. < 9 mill/mL, b. ≥ 9 mill/mL
- Baseline serum AMH: a. < 50 pmol/L, b. ≥ 50 pmol/L
- Baseline sperm production and serum AMH: a. < 9 mill/mL and < 50 pmol/L, b. < 9 mill/mL and ≥ 50 pmol/L, c. ≥ 9 mill/mL and < 50 pmol/L, d. ≥ 9 mill/mL and b. ≥ 50 pmol/L
- Baseline serum RANKL: a. Lowest tertile, b. Middle tertile, c. Highest tertile.
- Baseline serum OPG: a. Lowest tertile, b. Middle tertile, c. Highest tertile.
- Testicular size: a. average size < 20 mL, b. average size ≥ 20 mL
- Baseline serum PTH: a. Lowest tertile, b. Middle tertile, c. Highest tertile.
- Varicocele or cryptorchidism, a. yes, b. no
- BMI: (weight (kg)/ height^2^ (cm^2^) a.< 25, b. 25-30, c. >30

**Harms**We will report the total number of serious adverse reactions, as defined in the protocol for each group, as well as the total number of participants who experienced one or more serious adverse reactions in each group. We will also report the total number of serious adverse events, as defined in the protocol in each group, as well as the number of participants who experienced one or more serious adverse events in each group.

**Data integrity**

Prior to the analyses, the integrity of trial data will be checked by scrutinizing data files for omissions and errors. The source of any inconsistencies will be explored and resolved. To maximize data utilisation and improve patient care, the trial data will be made available via clinicaltrials.gov. once primary and secondary analyses have been published.

**Figures and tables**The first table will be the baseline characteristics of the ITT population. The second table will include the primary and secondary outcomes according to the two allocation and pairwise comparisons. The first figure will be a consolidated standard of reporting of randomized trials (CONSORT) flow chart. The following figures will include a rate of effect by treatment arms and also a plot illustrating all the preplanned sub analyses.  **Statistical software**All data will be entered as an electronic database in REDcap. The system is offered to researchers in the capital region of Denmark. Our data is thus protected by security code and only accessible for registered users. All statistical calculations will be conducted using SPSS version 25 and and R package version 3.4.1.

**References**

1. Boivin J, Bunting L, Collins JA, Nygren KG. International estimates of infertility prevalence and treatment-seeking: potential need and demand for infertility medical care. Hum Reprod. 2007;22(6):1506–12.

2. Lunenfeld B. Infertility in the third millennium: implications for the individual, family and society: Condensed Meeting Report from the Bertarelli Foundation’s Second Global Conference. Hum Reprod Update. 2004;10(4):317–26.

3. Huynh T. Selected genetic factors associated with male infertility. Hum Reprod Update. 2002;8(2):183–98.

4. Krausz C. Male infertility: Pathogenesis and clinical diagnosis. Best Pract Res Clin Endocrinol Metab. 2011;25(2):271–85.

5. Association WM. World Medical Association Declaration of Helsinki: Ethical Principles for Medical Research Involving Human Subjects. JAMA. 2013 Nov 27;310(20):2191–4.

6. ICH GCP - ICH harmonised guideline integrated addendum to ICH E6(R1): Guideline for Good Clinical Practice ICH E6(R2) ICH Consensus Guideline - ICH GCP.

7. Dwan K, Altman DG, Arnaiz JA, Bloom J, Chan A-W, Cronin E, et al. Systematic Review of the Empirical Evidence of Study Publication Bias and Outcome Reporting Bias. PLoS One. 2008;3(8):e3081.

8. Thomas L, Peterson ED. The Value of Statistical Analysis Plans in Observational Research. JAMA. 2012;308(8):773.

9. Gabriel SE, Normand S-LT. Getting the Methods Right — The Foundation of Patient-Centered Outcomes Research. N Engl J Med. 2012;367(9):787–90.

10. Gamble C, Krishan A, Stocken D, Lewis S, Juszczak E, Doré C, et al. Guidelines for the Content of Statistical Analysis Plans in Clinical Trials. JAMA [Internet]. 2017;318(23):2337. Available from: http://dx.doi.org/10.1001/jama.2017.18556

11. Demets DL, Lan KKG. Interim analysis: The alpha spending function approach. Stat Med. 1994;13(13–14):1341–52.

12. Groenwold RHH, Moons KGM, Vandenbroucke JP. Randomized trials with missing outcome data: how to analyze and what to report. Can Med Assoc J. 2014;186(15):1153–7.
